# GFTrans: an on-the-fly static analysis framework for code performance profiling

**DOI:** 10.3389/fdata.2026.1779935

**Published:** 2026-02-27

**Authors:** Jie Li, Yunbao Wen, Jingxin Liu, Biqing Zeng, Seyedali Mirjalili

**Affiliations:** 1 School of Artificial Intelligence, South China Normal University, Foshan, China; 2 School of Mathematics and Physics Sciences, RI-IM·AI*, Chongqing University of Science and Technology, Chongqing, China; 3 Center for Artificial Intelligence Research and Optimization, Torrens University Australia, Brisbane, QLD, Australia

**Keywords:** code representation learning, control flow and data flow, graph linearization, on-the-fly profiling, performance prediction, static analysis

## Abstract

Improving software efficiency is crucial for maintenance, but pinpointing runtime bottlenecks becomes increasingly difficult as systems expand. Traditional dynamic profiling tools require full build-execution cycles, creating significant latency that impedes agile development. To address this, we introduce GFTrans, a static analysis framework that predicts c program performance without execution. GFTrans utilizes a Transformer architecture with a novel “anchor-based embedding” technique to integrate control flow and data dependencies into a unified sequence. Additionally, a dynamic gating mechanism fuses these semantic representations with 16 handcrafted statistical features to comprehensively capture code complexity. Evaluated on a dataset of real-world GitHub c functions with high-precision runtime labels, GFTrans outperforms baseline models like Random Forest and Code2Vec, achieving 78.64% accuracy. The system identifies potential bottlenecks in milliseconds, enabling developers to perform optimization effectively during the coding phase.

## 1 Introduction

As software engineering is advancing rapidly, software systems have a strong impact on both user satisfaction and ongoing maintenance costs (Jafari Navimipour and Soltani, 2016). As software applications grow larger in size, it becomes more difficult for programmers to improve performance. Many programmers cannot easily pinpoint the cause of runtime slowdowns. Their dependence on latency-laden loops, inefficient memory access, and I/O (input/output) operations (Laaber et al., 2021) that block the line of code in a large software system makes this task harder. Traditionally, performance concerns are detected by the performer's analytical methods. Some tools, including Intel VTune (Intel, Santa Clara, CA, USA) and Gprof, monitor the runtime behavior of a particular application. These tools accurately determine where performance-related bottlenecks may exist. All of these methods require an ex post facto analysis.

To generate performance measurements, the developer must complete the full coding and building cycle. This includes creating detailed test cases, compiling the software, and running it in a representative sample environment. This type of “dynamic” analysis is an impediment to agile software development (Altuwaijri and Ferrario, 2022). When programmers have to interrupt their programming workflow to complete the compilation process, maintain a test environment, collect and analyze profiling data, and then return to their editor to make code changes, it results in wasted time and resources. This process limits the programmer's ability to receive timely feedback on performance issues. As a result, performance concerns are often not identified until testing or after deployment, resulting in expensive fixes. This problem has created an urgent need to develop and deploy On-The-Fly Performance Profiling (OTP). More and more studies have proposed code analysis frameworks for real-time analysis (Biringa and Kul, 2024). OTP enables programmers to predict the execution time of the code they are writing. It helps identify possible performance bottlenecks during code writing, without requiring the code to be run.

Predicting the complexity of a program's execution time based on its static source code attributes is a highly complex issue. The execution time of a program depends on both its control and data dependencies. The Control Flow Graph (CFG) and the Data Flow Graph (DFG) represent the performance characteristics of source code. These graphical representations illustrate the program execution paths, including branches and loops for CFG, and variable lifetimes and data dependency mechanisms for DFG (Cummins et al., 2021). Recent studies (Ma et al., 2023; Zou et al., 2025) find that the source code's graph structure can better capture the long-range relationships among a program's data. However, using graph network models to learn these structures often leads to significant model training costs (Huang et al., 2024). This high cost arises from the recursive message-passing mechanism on irregular graph structures. Unlike standard matrix operations, GNNs must iteratively aggregate features from neighbors, a process that is difficult to fully parallelize. Consequently, computational overhead grows exponentially as the code's complexity increases.

To address the aforementioned critical issue, a static analysis framework named Graph and Feature Transformer for Code Execution Time Prediction (GFTrans) is proposed to accurately predict the runtime duration categories of C programs. It combines semantic information from CFG with manually created code features. We developed an “anchor-based embedding” technique that merges CFG and DFG data from the C program into one linear sequence using explicit anchor tags. This unified structure enables Transformer-style architectures to utilize self-attention mechanisms, capturing both the execution flow and data dependencies within the program. Our self-created dataset categorizes codes into four types of execution time labels (from extremely short to long), and uses the GRTans model for predicting. Experimental results demonstrate that our model outperforms a series of classic baselines in terms of accuracy. Furthermore, we developed a code performance bottleneck detection system by embedding the trained model as the backend inference engine, enabling a “On-The-Fly” workflow for developers.

In a nutshell, the contributions of this research are as follows:

- We propose a static analysis framework named Graph and Feature Transformer for Code Execution Time Prediction (GFTrans) to accurately predict the runtime duration categories of C programs.
- We developed an ”anchor-based embedding” technique that merges CFG and DFG data from the C program into one linear sequence using explicit anchor tags. This unified structure enables Transformer-style architectures to utilize self-attention mechanisms, capturing both the execution flow and data dependencies within the program.
- We constructed a large-scale dataset derived from industrial-grade open-source projects. Extensive comparative experiments demonstrate that GFTrans achieves a better accuracy than a series of popular method. Our model attains 78.64% accuracy, exceeding the best baseline performance by 2.5 and 2.3%, respectively.

The rest of this paper is organized as follows. Section 2 reviews the related work on code performance prediction and graph representation of code. Section 3 presents the design of our GFTrans framework. Section 4 discusses the evaluation of our methodology, including the construction of our high-precision C function benchmark dataset, the experimental setup, baseline models for comparison, and evaluation results. Section 5 discusses the internal mechanisms of the model, its limitations, and use cases in IDEs. Finally, Section 6 concludes this paper and outlines future research plans.

## 2 Related work

This study focuses primarily on two different areas: code performance prediction and assessment, learning graph-based code representations. In the following sections, examples of the work that has been carried out within these two areas are examined, including how this study differs and advances upon what has already been done.

### 2.1 Code performance prediction and assessment

Traditionally, the analysis of code performance has been based on profiling techniques that rely on tools such as Gprof (Graham et al., 1982) and Intel VTune (Intel, Santa Clara, CA, USA) (Reinders, 2005). These toolsets rely on a full build-execution cycle to provide accurate performance data. This process is inherently lengthy and hinders the agile “write and test” workflow. However, nowadays, numerous researchers have investigated methods for developing predictive models of code performance utilizing data-driven learning techniques. For example, Mendis et al. (2019) described the first framework for predicting code execution throughput based on the basic blocks used in code execution, utilizing LSTMs to represent the throughput value for each basic block. The results of this research indicate that deep learning techniques are superior to more traditional models, such as LLVM-MCA, when it comes to simulating the efficiency of executing CPU instructions. However, Ithemal limits its predictions to the assembly code level, and therefore, cannot effectively capture more complex control flow structures that are present in higher-level languages. At the system and software levels, Weber et al. (2021) proposed a model architected to estimate the performance of software systems based on a combination of lightweight dynamic profiling data and structural features of the software code itself, hence the “white-box” nature of the model. This study concludes that exploring the internal structures of the code yields better performance predictions compared to using only black-box learning techniques. DeepPerf (Ha and Zhang, 2019) incorporated a deep sparse neural network model into its design to mitigate sparsity challenges in high-dimensional configuration space. Perf-AL (Shu et al., 2020) utilized adversarial learning techniques to augment the robustness of performance prediction models by utilizing models based on adversarial learning methodologies. Zheng et al. (2021) created TenSet as a means of predicting the execution time of underlying operators by training XGBoost and GNN on large-scale tensor program datasets. Sikka et al. (2020) demonstrated that the depth of nested loops and the number of branches in the code structure are important indicators of the computational efficiency of a code base.

Each of these approaches either emphasizes assembly or operator-based operations too much, or depends on dynamic runtime profiling data. GFTrans provides a benefit because of its ability to make static predictions. We only perform inference based on the source code graph structure and code characteristics, without compiling or executing the code. In this way, GFTrans provides millisecond-level feedback, filling the void created by the lack of a method for real-time performance analysis during software development.

### 2.2 Learning graph-based code representations

Source code naturally possesses a graph structure. Common representations include Abstract Syntax Trees (AST), Control Flow Graphs (CFG), and Data Flow Graphs (DFG). The main challenge lies in efficiently encoding these structural features into neural networks.

Early research, such as Code2Vec (Alon et al., 2019), focused on the “tree structure” of code. This work demonstrated that identifying pathways within the code is crucial for learning its semantics. Zhou et al. (2019) introduced the Devign. that combines AST, CFG, and DFG into a unified graph and uses Graph Neural Networks (GNNs) to detect vulnerabilities.

However, standard graph models are often incompatible with pre-trained models like BERT. To bridge this gap, Guo et al. (2020) introduced GraphCodeBERT, the first model to incorporate Data Flow Graph (DFG) structures during pre-training, thereby enhancing variable alignment. Building on the need to capture data flow semantics, Wang et al. (2021) developed CodeT5, which employs identifier-aware pre-training tasks. Additionally, to address syntactic structures, Guo et al. (2022) proposed UniXcoder, which effectively linearizes Abstract Syntax Trees (AST) into unified sequences.

Our GFTrans differs from these approaches. Unlike Devign, which relies on computationally heavy GNNs, GFTrans utilizes the efficient Transformer architecture. We draw inspiration from UniXcoder but specifically focus on the CFG to represent execution behavior. Crucially, we introduced “anchor tags” to integrate DFG data. These tags align data dependencies with the CFG execution pathway. This design allows the model to capture dynamic execution logic efficiently.

## 3 Methods

**Figure 1:**
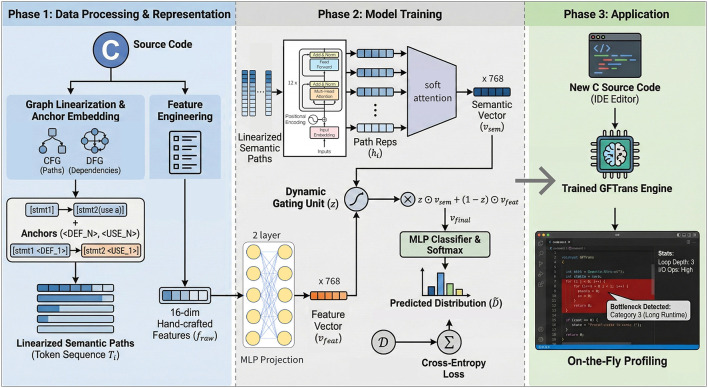
The overall architecture of GFTrans.

In this section, we design the GFTrans architecture (Graph and Feature Transformer for Code Execution Time Prediction) as presented in Figure 1. The GFTrans framework consists of three main phases: Data Processing and Representation, Model Training, and Application.

**Phase 1: data processing and representation**. We utilize c source code using two types of graphs: Control Flow Graphs (CFG) and Data Flow Graphs (DFG). we extract the execution logics paths from CFG. We then incorporate data dependencies from DFG into these execution paths by embedding anchor markers directly within the sequence of code statements. However, a solely path-based method of obtaining deep semantic features may not provide adequate information to capture specific, explicit structural metrics, which can significantly effects on execution time, including the number of loops nested and the frequency of types of operations executed. Therefore, We introduce a 16-dimensional handcrafted feature vector to capture keywords and execution structures. These features work alongside the CFG and DFG to enhance the model's semantic understanding.

**Phase 2: model training**. We firstly encode the serialized CFG and DFG using a Transformer Encoder and fuse multiple representation vectors through the attention mechanism to obtain our deep semantic representation vectors. Then, we also implement a Dynamic Gating Fusion Mechanism, which fuse the deep semantic feature vector generated by the Transformer with explicit handcrafted code feature vectors, fully utilizing their different contributions dynamically based on the input code characteristics. Finally, we predict the execution time level (extremely short, short, medium, or long) for the code using fully connected layers.

**Phase 3: application**. The output of the trained GFTrans accept new C source input and predict the execution time level in real-time, allowing developers to quickly identify performance issues occurring in their code.

### 3.1 Data processing and representation

We elaborate on the details of the code data processing and representation stages from two aspects: Graph Linearization and Anchor Embedding and Features Engineering.

#### 3.1.1 Graph linearization and anchor embedding

Source code is a non-linear representation, which contains a variety of complex structural information. When the DFG and CFG structural representations are used to create a model's understanding of code, it makes a significant improvement in the model's ability to understand the semantics of the code. Although GraphCodeBERT (Guo et al., 2020) incorporates Data Flow Graphs (DFG), it uses them implicitly during pre-training for variable alignment. In contrast, GFTrans employs an explicit “anchor-based” strategy. We inject discrete 〈*DEF*〉 and 〈*USE*〉 tags directly into the token sequence. This preserves precise define-use chains and forces the attention mechanism to actively track data dependencies during runtime prediction, ensuring a stronger grasp of code logic. One of our key challenges is the effective conversion of code graph structures into a linear representation that can be processed by Transformer-type architectures, without losing the underlying logical structure of the code or its data dependency. To address this, we present a linearization of the graph structure using dedicated anchor markers. this strategy is implemented in four steps.

Step 1: By using the static code analysis tool Joern, we are able to create a Code Property Graph (CPG) for the C function target being analyzed, from which we can identify three types of code collections. (1) Statement Node Set V: it contains all statement-level nodes and the associated source code that is represented. (2) Control Flow Edge Set E_CFG_ : a directed edge set that shows the relationships of execution jumps between statements within a CFG format. (3) Data Dependency Edge Set E_DFG_: a directed edge set that defines the “Def-Use” relationship chains, allowing us to ascertain all statement nodes defining data and usage statement nodes within a specific application process.

Step 2: To ensure ease of recognition when navigating through multiple code basic blocks of a series, a global indexing system be implemented to represent data segments within the code throughout the *E*_DFG_ set. We iterate through all data definition locations, and when we identify a data definition node in *V* (*v*_def_∈*V*), we assign it a unique numeric identifier ID(*v*_def_) = *N* (where *N*∈{1, 2, …, *M*} and *M* denotes total data definitions found in the function); this unique ID is used as a reference when accessing the data in the code tokens.

**Figure 2:**
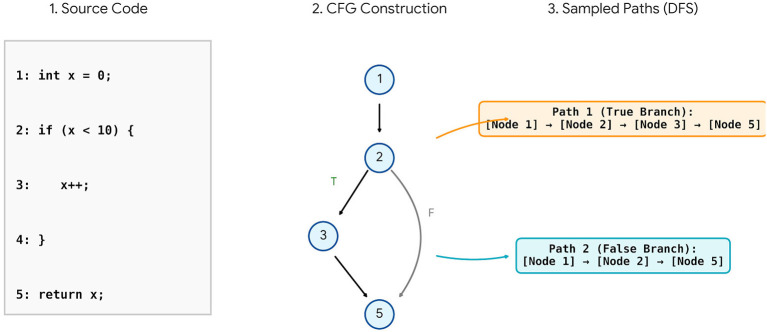
The process of extracting execution paths from the control flow.

Step 3: Figure 2 illustrates that the program launches in sequential order as defined by the execution of each of the statements. The networkX library provides an API to generate an control flow graph structure using python. Using a *Depth-First Search* (DFS) Technique, we can sample a CFG via traversal from the starting point to the end point, thus creating a Path where every statement Node encountered during the traversal path for a defined statement node is recorded. As a CFG may contain cycles, we track the number of times a statement node has been visited, limiting it to a maximum of two visits. This technique, therefore, allows traversing the cycle and reaching an endpoint without creating a continuous Loop. As we construct paths from the CFG , any code fragment results in a Collection of Execution Paths. Therefore, the Set of Paths Created from Within the CFG Graphs are Defined as $P=\left\{P_{1},P_{2},\ldots ,P_{K}\right\}$ where *K* = number of paths established. Each path *P* consists of a List of Statement Nodes; And *P* = [*s*_1_, *s*_2_, …, *s*_*L*_] and *s*_*i*_ = the *i*-th Statement Node in the Path. The arrangement of code statement sequences according to the control flow path reflects how the program is executed.

**Figure 3:**
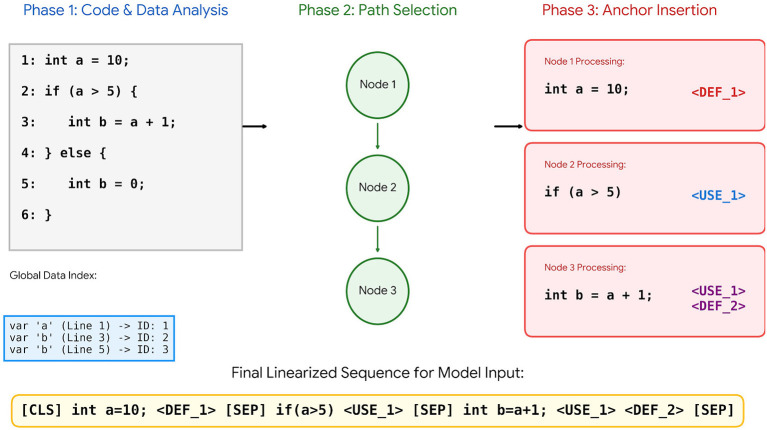
Schematic of the explicit anchor marker insertion strategy.

Step 4: Figure 3 illustrates the process of inserting data anchors into the code execution path. This embedding technique represents a key innovation of our work. Specifically, we develop a method to extract discrete DFG data and seamlessly incorporate it into continuous code execution sequences. For each statement node *s*_*i*_ in the sampled path *P*, information from the *E*_DFG_ is retrieved and structurally augmented to the execution path sequence of *s*_*i*_ by the addition of specially structured markers, as follows. For example, if the statement node *s*_*i*_ represents a data definition point and there exists a parent data anchor ID assigned to *s*_*i*_ in step 2 identifier *N*_ID_, we embed a unique definition marker tag  <DEF_{*N*_ID_}> into the textual content of statement Node *s*_*i*_. If *s*_*i*_ uses one or more data types specified from data definition point anchors in the *E*_DFG_, we locate all data definition point anchor IDs associated with *s*_*i*_ and will append the appropriate Number of use tags  <USE_{*N*_1_}>,  <USE_{*N*_2_}> into the textual representation of the statement node *s*_*i*_.

After enhancement, the path set retains the semantics of its original execution flow. However, explicitly defined data flow markers within the path can be combined with the separator label [SEP] for utilization in the final execution path sequence. In order for the model to recognize the data structure embedded in the execution path, All unique anchor Tags (e.g.,  <DEF_1>,  <USE_5>) must also be available to the word vocabulary of the pre-trained CodeBERT Model. After tokenization, truncation or padding the sequence to a pre-determined Length, the sequence must then be transformed into a fixed-length token ID sequence. The result is a guidance input to the model, which facilitates the full integration of control flows and data flows.

#### 3.1.2 Features engineering

The Transformer-based deep learning approach has demonstrated the ability to comprehend the semantics of code (Vaswani et al., 2017). Utilizing various code features allows an analyst to evaluate the complexity of a code base and allows the deep learning model to attain the most relevant inductive biases, based upon a larger scope of coding features. This research uses the feature set developed by Sikka et al. (2020), who produced a set of features based upon statistical analysis techniques to assist in the development of models to predict the runtime complexity of code. We implemented a total of 24 features using SourceMonitor tools based on source code generation, along with custom-written scripts to develop a 24-dimensional feature vector. These features are categorized across four distinct structural complexity classes.

Sikka et al. (2020). demonstrated that the depth of nested loops and the number of branches in the code structure are important indicators of the computational efficiency of a code base. Therefore, we has produced a total of 24 features based upon source code using a tool named SourceMonitor, along with custom written scripts to develop a 24-dimensional feature vector; these features are based upon four different categories of structural complexity.

The structural complexity features category includes items such as nested loop depth, number of Loops, number of Ifs, number of Switches, and maximum Block depth. The count of methods category includes IO keyword counts, counts of total statements, counts of total variables, and counts of total code lines, as well as the high overhead costs associated with executing IO calls. The algorithms patterns category includes the number of sorts associated with sorting, whether recursion is present, whether a priority queue is present, whether a hash map is present, and a variety of other elements.

Lastly, the control flow fragmentation characteristics category includes the total number of jumps, breaks, continues, and returns to illustrate the extent to which the execution of a program has deviated from its intended path. The original 24-dimensional features developed in this research have the potential to contain noise or redundancy. Using Spearman rank correlation, we investigat the correlation between code features and run times of the code in the training set. From the training set labels, we calculate the Spearman Correlation Coefficient ρ to determine monotonic relationships between feature values and runtime of the code.

**Figure 4:**
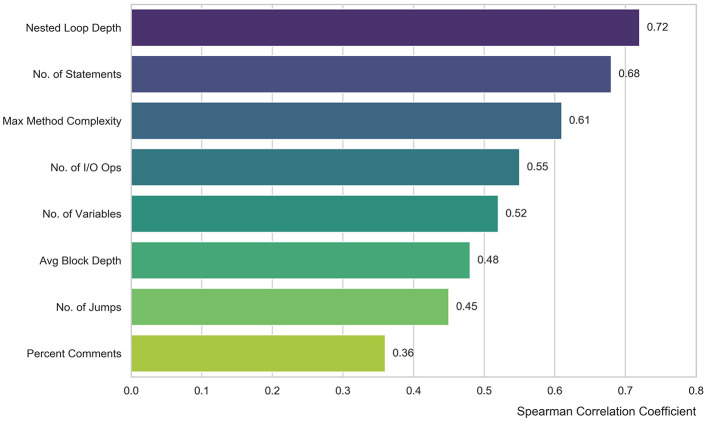
Top eight handcrafted features ranked by Spearman correlation.

To empirically validate the necessity of these handcrafted features, we calculated the Spearman rank correlation coefficients for all 24 features against the runtime labels and plotted the top 8 most influential features in a bar chart (Figure 4). As expected, structural features rank the highest, with “Nested Loop Depth” (0.72) and “Number of Statements” (0.68) demonstrating the strongest positive correlation. This confirms that algorithmic complexity, driven by loops and code size, is the primary determinant of execution time. “Number of I/O Operations” also shows a strong correlation (0.55) due to the inherent latency of I/O calls. Interestingly, while “Percent Comments” has a lower correlation of 0.36 (below the 0.4 threshold), we retained it intentionally as a semantic proxy to assist the model distinguish between complex human-written logic and simple auto-generated code.

We specifically included “Percent Lines with Comments” as a key feature. Comments are stripped away by the compiler. They do not physically slow down the execution.We included this feature as a semantic indicator. The decison is grounded in the coding conventions of open-source communities. Firstly, developers tend to write detailed comments for complex algorithms. These algorithms usually fall into the “Long” execution category. Therefore, this feature acts as a “helper.” It helps the model estimate the complexity of the logic. we delete the Line feature and retained only the statement feature, which is better representative of how many statements are in each piece of code. Thus, the feature set has been reduced from 24 dimensions to 16 features of superior quality, as shown in Table 1.

**Table 1:** Description of selected features for static analysis.

**Feature name**	**Description**
nested_loop_depth	Depth of nested loops
Maximum Method Complexity	Cyclomatic complexity
Average Block Depth	Average nesting depth of code blocks
no_of_ifs	Count of if statements
no_of_switches	Frequency of multi-way branch structures
noOfJumps	Count of jump statements (break, continue, return)
no_of_io	Number of system I/O operations
Statements	Total number of effective instructions
noOfMethods	Frequency of function/method calls
noOfVariables	Total number of variables declared
recursion_present	Presence of recursion flag
no_of_sort	Count of sorting algorithm invocations
priority_queue_present	Presence of priority queue (Heap) operations
hash_map_present	Usage of hash-based data structures
hash_set_present	Usage of set-like logic or uniqueness checks
Percent lines with comments	Ratio of comments to code (Code quality metric)

### 3.2 Model training

The most significant challenge associated with training the model is the need to combine two types of multimodal data sets: a graphical structure with unstructured pathways and a set of handcrafted explicit features that are determined statistically (namely the 16 handcrafted explicit features). The two types of multimodal data sets needed to be integrated into one framework. To do that, we create a Modular Network that contains a variety of mechanisms to correlate the two types of multimodal data sets, including path segmentation, CodeBERT semantic encoding, nonlinear feature mapping, and dynamic gated fusion.

#### 3.2.1 Path segmentation and CodeBERT semantic encoding

By employing the CFG, we generated a collection of Paths $P=\left\{P_{1},P_{2},\ldots ,P_{K}\right\}$, where each path contains a traverse of all the properties of code and the *Anchor Points* representing their flow of data, using the edges associated with every path. The procedures employed to produce each dimensionality reduction vector are consistent with each of the paths that are sampled (i.e., *K* = 10). Initially, the CodeBERT pre-trained tokenizing method is utilized to convert each *P*_*i*_ (the *i*-th sampled path) into an array of sub-word token IDs. Simultaneously, we assign a unique anchor ID (i.e.,  <DEF_N>,  <USE_N>) to each of the IDs associated with each path. The final representation is denoted as *T*_*i*_.

Subsequently, *T*_*i*_ is processed by the backbone network, where tokens in *T*_*i*_ capture long-distance relations through self-attention within the Embedding layer and the 12-layer Transformer encoder. The resultant output vector aligns with the input representation at position [CLS]. Thus, the semantic representation of an individual path *h*_*i*_ is created as follows:


$$\begin{array}{c} h_{i}=\text{Encoder}\left(T_{i}\right),\;h_{i}\in {\mathbb{R}}^{d_{model}} \end{array}$$ (1)

where *d*_*model*_ is equal to 768. After the process of dimensionality reduction is completed for each code sample, the *h*_*i*_ vectors form a collection of *d*_*model*_ dimensional vectors *H* = {*h*_1_, *h*_2_, …, *h*_*K*_}, where each vector represents a different execution flow and its associated data dependencies.

#### 3.2.2 Path aggregation via attention mechanism

Execution paths in programs affect performance differently (for example, execution paths within the main loop of an algorithm significantly impact performance compared to paths in exception handling or initialization). Therefore, basic average pooling fails to capture these crucial structural differences. Inspired by Hu et al. (2025) and their use of soft-attention in graph-level readout operations for GNNs, we introduce a path-level attention mechanism to dynamically aggregate multiple path features. This mechanism allows the model to automatically learn the importance weight of each path.

We introduce a trainable context query vector $u\in {\mathbb{R}}^{d_{model}}$. For each representation vector *h*_*i*_ within the path set *H*, we first calculate its importance score *s*_*i*_ through a nonlinear transformation, and then apply a softmax function to normalize the score, obtaining the attention weight α_*i*_ for the *i*-th path:


$$\begin{array}{c} s_{i}=\tanh\left(W_{att}h_{i}+b_{att}\right) \end{array}$$ (2)

$$\begin{array}{c} \alpha_{i}=\frac{\exp\left(s_{i}^{⊤}u\right)}{\sum_{j=1}^{K}\exp\left(s_{j}^{⊤}u\right)} \end{array}$$ (3)

where *W*_*att*_ and *b*_*att*_ are trainable parameters, and *K* is the total number of paths. The weight α_*i*_ quantifies the contribution of the *i*-th path to determining the current code's runtime category. The final code semantic vector *v*_*sem*_ is produced via the weighted sum of all path vectors:


$$\begin{array}{c} v_{sem}=\overset{K}{\underset{i=1}{\sum }}\alpha_{i}h_{i} \end{array}$$ (4)

This process compresses the *K*×768 path matrix into a single 1 × 768 global vector, effectively highlighting the semantic features of key execution paths while suppressing noise from irrelevant paths.

#### 3.2.3 Nonlinear projection of handcrafted features

In the feature engineering phase, we screen out a 16-dimensional high-quality handcrafted feature vector $f_{raw}\in {\mathbb{R}}^{16}$ (including nested loop depth, IO keyword frequency, etc.). Since the dimension of handcrafted features is far lower than that of semantic vectors and the numerical distribution varies significantly, direct concatenation would cause the explicit features to be overwhelmed by high-dimensional semantic features. Therefore, we design a two-layer Multi-Layer Perceptron (MLP) as a feature projection module. First, we perform Z-Score normalization on *f*_*raw*_ to obtain *f*_*norm*_, and then project it to the same dimension space as the semantic vector:


$$\begin{array}{c} v_{feat}=\text{GELU}\left(W_{2}\left(\text{GELU}\left(W_{1}f_{norm}+b_{1}\right)\right)+b_{2}\right) \end{array}$$ (5)

where $W_{1}\in {\mathbb{R}}^{16\times 64}$ transforms the features to an intermediate layer, and $W_{2}\in {\mathbb{R}}^{64\times 768}$ further maps them to the target semantic space. The projected $v_{feat}\in {\mathbb{R}}^{768}$ can then interact with *v*_*sem*_ in the same manifold space.

#### 3.2.4 Dynamic gated fusion and classification

To adaptively adjust the contribution ratio of deep semantic features and code features in the final prediction, we adopted a dynamic gating unit. The gating vector *z*∈(0, 1)^768^ is generated by jointly analyzing both feature streams:


$$\begin{array}{c} z=\sigma \left(W_{g}\left[v_{sem};v_{feat}\right]+b_{g}\right) \end{array}$$ (6)

The final fused vector *v*_*final*_ is computed via element-wise multiplication (⊙). To ensure dimensional consistency, the handcrafted feature vector is projected to match the semantic vector's dimension, so $v_{sem},v_{feat}\in {\mathbb{R}}^{768}$. The gating vector *z* is also generated in ℝ^768^. The fusion equation is defined as:


$$\begin{array}{c} v_{final}=z⊙v_{sem}+\left(1-z\right)⊙v_{feat} \end{array}$$ (7)

where ⊙ represents element-wise multiplication. This mechanism allows the model to preserve deep semantics in certain dimensions (such as capturing data flow dependencies) while directly utilizing explicit features in others (such as exponential complexity signals brought by loop depth). Finally, the fused feature vector *v*_*final*_ is input to the classification head, which consists of a fully connected layer and a Softmax activation function, outputting the probability distribution of four runtime categories:


$$\begin{array}{c} P=\text{Softmax}\left(W_{c}v_{final}+b_{c}\right) \end{array}$$ (8)

The model training uses the standard Cross-Entropy Loss function:


$$\begin{array}{c} L=-\frac{1}{N}\overset{N}{\underset{i=1}{\sum }}\overset{3}{\underset{c=0}{\sum }}y_{i,c}\cdot \log\left(P_{i,c}\right) \end{array}$$ (9)

where *N* is the batch size, *y*_*i, c*_ is the true label of sample *i* belonging to runtime category *c*, and *P*_*i, c*_ is the predicted probability.

To provide a holistic view of the proposed framework, we formalize the complete training procedure in Algorithm 1.

Algorithm 1 GFTrans training pipeline. 
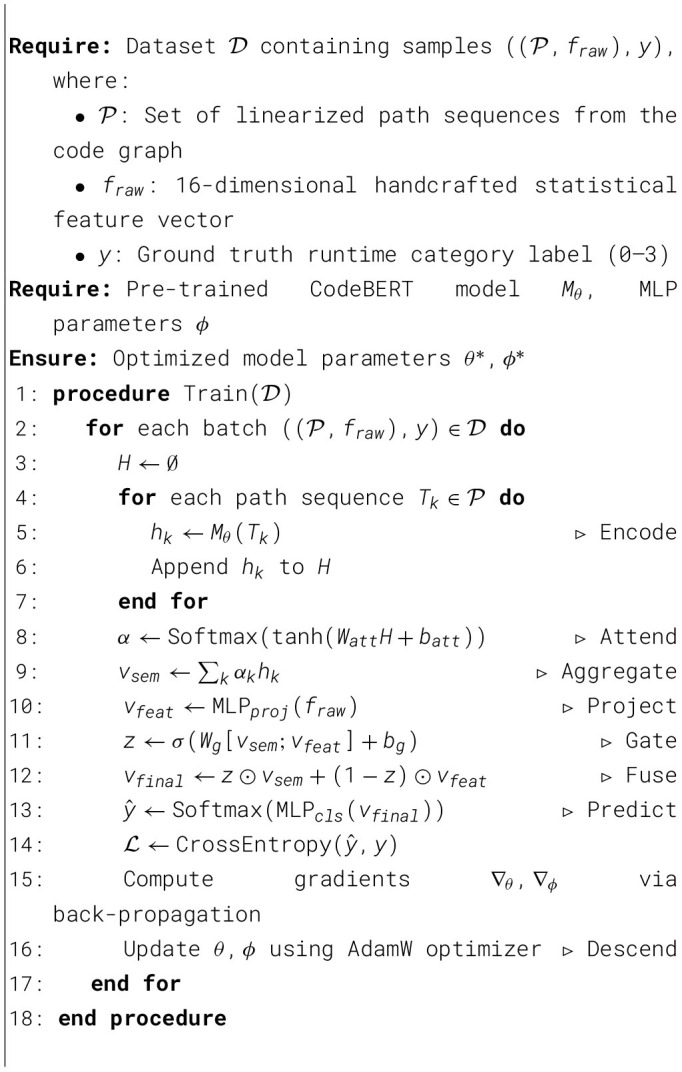

### 3.3 Application

Utilizing the proposed GFTrans model, we implemented an automation system that analyse code for performance bottlenecks. This system provides developers with feedback using “time profiling” tools through static analysis methods that help identify code quality enhancements by quickly identifying any potential high-cost problems during coding. The primary function of this system is to create a trained GFTrans model, which acts as an inference engine and is used in contrast to dynamic performance analysis utilities, which require compilation, instrumentation or running of test cases. Instead, this system performs inference only on the source code attributes. The processing stages of this system can be described in three phases:

• Automated parsing/extraction: after saving the C source file(s), the system generates a parser, which automatically divides the code into individual function segments. Each function will also simultaneously allow the retrieval of its graph structure (CFG/DFG) path sequence with a total of 16 custom statistical features manually created.

• Complete model inference: the multimodel features retrieved from the parser automatically feed into the trained GFTrans model. The model comprehensively evaluates control logic and data dependencies to generate a probability distribution. This distribution represents the likelihood of the function falling into one of four runtime categories: 0 (Extremely Short), 1 (Short), 2 (Medium), and 3 (Long). The last category is identified as the predicted class.

• Feedback generation/display: after identifying “Long-running” code blocks or functions, the system creates a red highlighting background in the code. This visual representation provides the developer a way to quickly identify the potential performance bottleneck when reviewing or writing their code, which eliminate the potential for inefficient code to be deployed into production environments. Clicking the highlighted function will display statistical details of that block of code in the sidebar.

To integrate seamlessly into the standard developer workflow, the system is designed as an IDE extension. This extension, which leverages the GFTrans model's graph handling capabilities, provides real-time feedback while the developer is coding, thereby allowing immediate feedback without having to set up complicated compilation environments or generate test case inputs. By moving performance evaluation from “post-mortem” to “prevention” the system significantly reduces the difficulty of performance evaluations and provides both a tool for engineers to optimize their performance, as well as a learning tool for new developers to assist them in correcting inefficient coding patterns on the spot.

## 4 Evaluation

### 4.1 Data preparation

This section details the process of developing a runtime benchmark dataset for C programming. Unlike research projects focusing on complete source code, our dataset concentrates on function-level code snippets. This fine-grained dataset enhances the precision of runtime prediction. It also fulfills the industry's need for diagnosing performance issues based on individual functions.

**1) Data collection:** most public datasets come from various algorithm competition platforms. However, the programming styles in competitive programming from those found in practical software development processes. To ensure our datasets have broad diversity and represent real-world coding styles, we select our dataset from the GitHub. We sample from 300 updated, publicly available C open source projects, each with over 300 stars, containing a wide range of coded works. These include low-level system utilities, high performance network libraries, and data processing algorithms. Code from GitHub repositories better represents user practices and coding styles found in industry than current open source datasets. Collectively, we amasse a total of 181 GitHub-sourced open source projects containing C code.

**2) Data filtering and preprocessing:** we filter the data through scripts to ensure that the retained code snippets cover all complex code structures, including branches, loops, memory allocation, IO operations, etc. This diversity of code structures helps the model generalize better. On the other hand, we remove overly simple and overly lengthy code snippets, such as single-line code snippets and code snippets exceeding 200 lines. To comply with the Transformer's 512-token limit, we applied a strict length filter during preprocessing. We pre-calculated the total length of each function, accounting for the additional anchor tags (〈*DEF*〉, 〈*USE*〉). Any sample exceeding this limit was excluded. This proactive filtering ensures that all samples in our dataset are processed in full without truncation, preserving the complete integrity of the control flow and data dependencies. Finally, to exclude code with syntax errors, we only retain code that compiles without errors among these code snippets.

**3) Code runtime collection:** we run the code in a unified environment to collect the running duration of the code. All operations are performed on a computer equipped with an Intel Core i7-12700H CPU @ 2.30GHz and 32GB of memory, with the operating system being Ubuntu 22.04 LTS. During the test, CPU scheduling is disabled, and only a single CPU core is used when running the code, which reduces the impact of operating system scheduling. All C code samples are compiled using GCC 11.4 with the -O2 optimization flag, which avoids the impact of compiler optimization techniques on execution time. For timing, we use Linux nanosecond timing to measure each function from start to end. Each sample run 20 times independently. We discard the fastest five and slowest five runs to remove hardware interrupt effects. To ensure the high quality of our dataset, we conducted a rigorous stability analysis. The operating system (Ubuntu) can introduce noise. This includes context switching and background processes. To mitigate this, we first applied a trimmed mean filter. We discarded the fastest 5 runs and the slowest 5 runs for each function.

Since the operating system (Ubuntu 22.04) is not a real-time system, background tasks. may introduce non-deterministic delays. Therefore, we employed the “trimmed mean” method by discarding the five fastest runs and the five slowest runs, which effectively removes outliers caused by system noise. To further address your concern, we analyzed the variance of the remaining 10 runs for each function using the Coefficient of Variation (CV). The results show that the CV is lower than 0.05 (5%) for 96.5% of the code samples. This low variance confirms that the execution time is highly stable. The assigned runtime category is robust.

**4) Statistics of the dataset and partitioning of the dataset:** after applying the sampling method to create our final dataset, we collect 2,887 highly-shielded C program examples. Based on execution time calculations, we divide the samples into four categories. We control sample category imbalance to less than 10% using undersampling and oversampling.

As indicated in Table 2, it is a summary we divide the execution times of the samples into four categories. The groupings of the samples on these four levels allow the prediction of runtime execution.

**Table 2:** Runtime execution categories and counts.

**Category**	**Runtime range**	**Count**
Ultra-short execution	≤ 20 ms	682
Short execution	>20ms and ≤ 100 ms	802
Medium execution	>100 ms and ≤ 500 ms	701
Long execution	≥500 ms	702

### 4.2 Baselines model comparison

To assess the capabilities and advantages of the GFTrans models in identifying runtime evaluation and best fit/cost performance for code execution classification, we compare the GFTrans results against those of three major baseline model categories. These categories represent the major methodologies and include:

- **Random forest:** a Random Forest model is widely used in software engineering studies for identifying development errors and estimating security-related bugs. It is highly accurate, reliable, and efficient. This study uses CodeBERT to show how Trained Models can utilize a Type BERT to generate 768-Dimensional Dense Vectors. These vectors represent the *Global Semantic Embedding* for Source Code. For input to Random Forest, we extracted them from the [CLS] Token output of the last CodeBERT layer. The Random Forest builds Decision Trees independently for each training instance. It estimates, in parallel, the Runtime Complexity Living Category for the input Code Samples. The output is determined by combining results from all Decision Tree predictions.
- **Long short-term memory (LSTM):** LSTMs are a widely used form of Recurrent neural network in software Engineering. They show how deep learning techniques can handle sequence data. For our assessment, we use the same vocabulary and embedding size of 768 as GFTrans. LSTMs can pass generated outputs back to the input layer using states, allowing efficient modeling of code's sequential token relationships. For classification, we use the final time step's state as the temporal representation when feeding into the last layer of a fully-connected model.
- **Code2Vec:** This is a model based on the structural features of code, using structure as the main representation. In this research, we follow a previously described approach to develop fixed-length feature vectors that capture code features from source code. We parse C source code into abstract syntax trees (ASTs) and generated “path contexts” to track structural characteristics. The model then combines these path characteristics into a complete semantic vector. This vector is fed into a classification layer, which predicts one of four categories based on the execution time of the C function. We use this as a baseline for comparing our graph-based method (CFG/DFG) to AST-based approaches.

### 4.3 Evaluation metrics

To quantitatively evaluate the accuracy of the predictive model, we create a confusion matrix from the model's predictions based on the execution times of C functions. The model's performance is evaluated using the following common classification evaluation metrics:

**Accuracy:** the ratio of all instances predicted by the model to the total number of instances predicted; this gives a simple measure of how well the classification model can be expected to perform across the population of instances.


$$\begin{array}{c} Accuracy=\frac{TP+TN}{TP+TN+FP+FN} \end{array}$$ (10)

where *TP*, *TN*, *FP*, and *FN* are defined as true positive, true negative, false positive and false negative respectively.

**Precision:** the number of samples identified as having a positive condition divided by the total number of samples assigned a positive condition; this measures the accuracy of the model's predictions. The precision for each class *i* is expressed as:


$$\begin{array}{c} Precision_{i}=\frac{TP_{i}}{TP_{i}+FP_{i}} \end{array}$$ (11)

**Recall:** recall refers to how many positive instances were correctly labeled positive by the model, and is the measure of how well the model can detect examples. The recall for each class *i* can be expressed as:


$$\begin{array}{c} Recall_{i}=\frac{TP_{i}}{TP_{i}+FN_{i}} \end{array}$$ (12)

**Macro-F1 score:** is less reliable than a standard accuracy metric, because the frequencies of different runtime categories vary greatly from category to category (i.e., very short samples could be more common than very long ones). Macro-F1 will compute the F1 score for each individual category, then average all of the F1 scores to provide a balanced view of the model's performance for all classes:


$$\begin{array}{c} Macro-F1=\frac{1}{C}\overset{C-1}{\underset{i=0}{\sum }}\frac{2\times Precision_{i}\times Recall_{i}}{Precision_{i}+Recall_{i}} \end{array}$$ (13)

where *C* = 4, Represents the total number of Classes. The Macro-F1 Score evaluates the precision and recall values together.

### 4.4 Experimental setup

We implement GFTrans and all baseline models using the PyTorch framework. To ensure the reproducibility of our results and fair comparisons, all experiments are conducted under the same hardware and software configurations. The detailed settings are as follows:

- **Hardware and software environment:** all models are trained and evaluated on a workstation running Ubuntu 22.04 LTS. The system is equipped with an Intel Core i7-12700H CPU @ 2.30 GHz and a single NVIDIA RTX 3090 (NVIDIA, Santa Clara, CA, USA) GPU (24 GB).
- **Model hyperparameters:** we align the hidden dimension size with the pre-trained CodeBERT model, setting it to 768. To prevent overfitting, we apply a dropout rate of 0.1 across the fully connected layers.
- **Training configuration:** we utilize the AdamW optimizer to update model parameters. The initial learning rate is set to 2 × 10^−5^, coupled with a linear learning rate scheduler to accelerate convergence. The batch size is set to 16. To accommodate GPU memory constraints while maintaining training stability, we implement gradient accumulation with a step size of 2.
- **Training strategy:** we employ an early stopping mechanism to save training time and prevent overfitting. Specifically, the training process terminates if the validation accuracy does not improve for 5 consecutive epochs, with the maximum number of epochs set to 25.
- **Validation strategy:** to ensure statistical reliability and minimize random variance, we employed a five-Fold Cross-Validation strategy. The entire dataset was randomly shuffled and divided into five equal folds. The training process was repeated five times. In each iteration, four folds were used for training and one fold for testing. The final reported metrics represent the average performance across these five independent runs.

### 4.5 Experimental results

In this section, we study the results to understand the relative effectiveness of the GFTrans.

#### 4.5.1 Evaluation of the GFTrans model's effectiveness

As shown in Table 3, the GFTrans model outperformed the leading baseline model (Code2Vec) by 2.5% points in accuracy and 2.3 percentage points in the F1 score. This indicates that the “CFG/DFG graph structure fusion” (herein referred to as “our approach”) produces additional improvement in prediction accuracy compared to other approaches, showing the value of following execution paths through the use of CFG and DFG data structures.

**Table 3:** Overall performance comparison between GFTrans and baseline models on test set.

**Model**	**Accuracy (%)**	**Macro-precision (%)**	**Macro-recall (%)**	**Macro-F1 (%)**
LSTM	73.58	72.15	70.88	71.45
Random Forest	74.82	73.80	72.18	72.93
Code2Vec	76.15	75.12	74.05	74.52
**GFTrans (Ours)**	**78.64**	**77.20**	**76.58**	**76.85**

Bold values indicate the best performance among all tested models for each metric.

The structural aspect of representation appears to have an advantage over unstructured forms of representation. The results indicate that models using code structure features (herein Code2Vec and GFTrans) produced more accurate prediction results than those based on flat features alone (Random Forest and LSTM). Code2Vec was able to outperform Random Forest by 1.3% points (Code2Vec = 76.1%, Random Forest = 74.8%), suggesting that hierarchical representations of code enable a better understanding of the logic contained within source code. The GFTrans model outperforms the Code2Vec model, it shows that compared to the static syntax tree features used by Code2Vec, dynamic control flow graph (CFG) and data flow graph (DFG) information are more efficient and accurate in predicting “runtime.”

In the case of sequential models (LSTM), the accuracy falls short compared to that of GFTrans (73.6%). The limitations of sequentially representing code are evident in the low LSTM accuracy. The nature of function calls and looping jumps creates long ranges of dependency that cannot be well learned by a simplistic sequential learning algorithm.

A comprehensive assessment of the efficacy of each model type across the runtime category is provided in Table 4. The experimental results indicate that all models exhibit relatively low prediction accuracy when predicting the two categories of “short” and “medium.” The accuracy of the baseline model hovers around 70%. The runtime differences between these two categories of code are not significant, with the main distinctions lying in the number of memory accesses, IO operations, and other time-consuming code statements. GFTrans achieves a runtime accuracy of 74% for these two categories, thanks to the introduction of manual code features that quantify the impact of time-consuming statements, thereby distinguishing minor performance losses caused by frequent memory operations.

**Table 4:** Detailed accuracy and F1-score for each model across four runtime categories.

**Category**	**Metric**	**Random forest**	**LSTM**	**Code2Vec**	**GFTrans (Ours)**
Ultra-short	Accuracy	80.52	79.45	81.24	**83.52**
	Precision	79.15	78.20	80.45	**82.10**
	Recall	77.38	76.89	78.68	**80.03**
	F1-Score	78.25	77.54	79.56	**81.05**
Short	Accuracy	69.85	68.52	71.55	**74.28**
	Precision	68.45	67.12	70.80	**73.55**
	Recall	66.65	65.32	68.86	**71.58**
	F1-Score	67.54	66.21	69.82	**72.55**
Medium	Accuracy	71.45	70.15	73.82	**76.54**
	Precision	70.50	69.25	72.15	**75.10**
	Recall	69.21	67.80	70.96	**73.42**
	F1-Score	69.85	68.52	71.55	**74.25**
Long	Accuracy	77.46	76.20	77.98	**80.22**
	Precision	77.10	74.03	77.08	**78.05**
	Recall	75.08	73.04	77.22	**81.10**
	F1-Score	76.08	73.53	77.15	**79.55**

The best results for each metric within each runtime category are highlighted in bold.

GFTrans achieves an accuracy of 80.2% and an F1 score of 79.5% for the “long-duration” category. This result highlights the effectiveness of our graph structure fusion strategy. In real-world scenarios, long-running programs often involve complex control flows and data dependencies. Baseline models tend to under-predict execution time because they treat code as simple sequences. In contrast, GFTrans fuses Control Flow Graphs (CFG) and Data Flow Graphs (DFG). This fusion enables the model to track long-range dependencies within deep loops and recursive calls. Consequently, GFTrans accurately captures the structural complexity that leads to long execution times.

**Figure 5:**
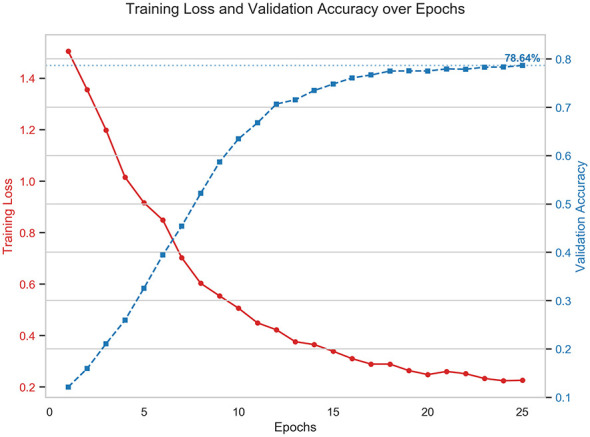
Training loss and validation accuracy.

To demonstrate the stability and convergence of our training process, we visualized the learning curve in Figure 5. Figure 1 depicts the Training Loss and the Validation Accuracy over 25 epochs. As shown in the figure, we observe the training loss (red line) decreases rapidly during the first 10 epochs, suggesting the GFTrans efficiency learns features from the code graph structure. The validation accuracy (blue line) rises quickly and stabilizes after Epoch 15. The accuracy fluctuates slightly around 78.6% without any significant drops. The gap between the training loss and validation accuracy remains reasonable, indicating the model generalizes well to unseen data. Furthermore, these curves justify our decision to use “Early Stopping.” Stopping the training at 25 epochs is sufficient to get the best performance without wasting computational resources.

**Figure 6:**
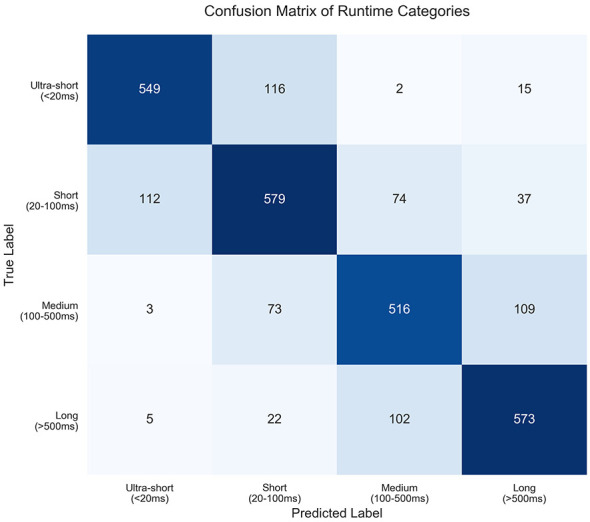
Confusion matrix of GFTrans predictions.

Furthermore, to better understand the nature of the misclassifications, we analyzed the results using a Confusion Matrix, as shown in Figure 6. The matrix reveals a clear and meaningful pattern. Firstly, the values on the diagonal are very high. This confirms the strong performance of GFTrans. Second, as you suspected, most errors happen between neighboring categories. For instance, the model sometimes confuses “Short” with “Medium.” But the model rarely makes severe mistakes. It does not confuse “Ultra-short” with “Long.” The errors mostly come from borderline cases. For example, a function runs in 98ms. The threshold is 100ms. The model might predict “Medium” (Category 2) instead of “Short” (Category 1). This is reasonable. Our calculation shows that about 88% of all misclassifications involve adjacent classes.

#### 4.5.2 The impact of components on GFTrans performance

To verify the intended design of GFTrans, each component of GFTrans was removed one at a time, and the impact on accuracy, F1 score, and overall accuracy was measured. The results of this study are displayed in Table 5.

**Table 5:** Ablation study results for different GFTrans variants.

**Model variant**	**Accuracy (%)**	**Macro-F1 (%)**	**Δ Acc**
w/o Hand-crafted features	77.14	75.38	–1.50%
w/o graph paths (Seq only)	75.12	73.45	–3.52%
Only CFG paths (w/o DFG)	77.25	75.52	–1.39%
w/o gating fusion	77.65	75.88	–0.99%
**GFTrans (full)**	**78.64**	**76.85**	–

Bold values denote the optimal performance, representing the full GFTrans model's results.

As shown in Table 5, removing graph-structured paths causes the accuracy to drop to 75.1%. This performance is lower than that of Code2Vec. This result validates our “data flow enhanced CFG linearization” component. It distinguishes GFTrans from prior work. Lexical co-occurrences alone are insufficient to determine program operations. They fail to capture control flow transitions that lack direct lexical relationships. Therefore, the directionality and structure of a CFG are essential. However, the CFG alone is not adequate. Comparing the CFG-only model with GFTrans reveals an accuracy gap of 1.4%. This increase comes from the addition of data flow paths. This supports the claim that control flow must be used with data flow. Together, they accurately determine the execution operation. This mechanism captures the definition and usage sequences of variables. Consequently, the model accumulates precise variable states to predict execution duration. We also evaluate the handcrafted features. Removing them from the network decreases accuracy by 1.5%. This indicates that these features introduce significant informative guidance. When code segments contain semantic ambiguity, these features act as “calibrators.” They provide rigid metrics to correct the model's predictions.

The plain concatenation of the feature vectors yielded a performance that was 1.0% lower than when the Gated Fusion method was employed. This suggests that code execution can be impacted by both the semantic and structural properties of the code. The Gated Fusion method allows for the model to selectively focus on the most informative features and eliminate those that are less informative. This enables the multimodal data to work together in a synergistic manner, allowing for adaptive synergy among the multimodal data.

#### 4.5.3 The effect of the compiler optimization level on generalization of the GFTrans model architecture.

Compiler optimization levels (GCC's -O0, -O2, -O3 and Os) have an impact on the relationship between program's characteristics and its runtime. The different levels of optimization produce machine code that creates a different set of runtime labels. To investigate the GFTrans model architectures' generality and flexibility under varying conditions of compilation, the experiments below were developed:

- **Dataset reconstruction:** for all projects in the test dataset, we did not modify the source and ran each using both the -O0, -O3 and -Os flags. As a result, this produced three unique sets of runtime labels.
- **Model retraining:** training and evaluation of the revised model for both -O0, -O3 and -Os Datasets, Using the Same Hyperparameters.

Table 6 summarizes the results of the above experiments. GFTrans achieves an accuracy of between 77 and 79% for either -O0 (preserving all redundant instructions) or -O3 (through intensive rearrangement of instructions). This indicates that the GFTrans graph-structured path and feature representation method proposed in this work is capable of generalizing robustly, allowing it to adapt to all compiler optimization conditions rather than exclusively overfitting to the unique characteristics of -O2. Finally, the accuracy of GFTrans under the -O3 condition (78.12%) is very close to that achieved under -O2 (78.64%). The fact that optimizations at the compiler level may have changed the quantity of executed instruction counts, but did not alter the time complexity class. It confirms that GFTrans captures the logical structure of the execution at a higher level using CFG/DFG, and is, therefore, the most robust against changes made by compiler optimization. In addition to speed optimizations (-O2, -O3), we also investigated the -Os flag, which optimizes for code size. Our supplementary test showed that GFTrans maintains a robust accuracy of 76.92% under -Os. The slight performance drop (compared to 78.64% under -O2) is expected, as -Os prioritizes smaller binary size over execution speed (e.g., by disabling loop unrolling), which can subtly alter the control flow structure used by our model.

**Table 6:** Impact of compiler optimization level on model generalization.

**Optimization level**	**Accuracy**	**Δ Acc**
-O2 (default)	78.64%	–
-O0	75.32%	–3.32%
-O3	76.85%	–1.79%
-Os	76.92%	–1.72%

## 5 Analysis and discussion

Following the quantitative evaluation of GFTrans, this section delves into the model's interpretability and practical applicability.

### 5.1 The complementary mechanisms within GFTrans through its dual feature set

The key benefit of GFTrans is the incorporation of “deep semantics,” as well as “explicit structural features.” To understand how the combined presence of these features is used by the model, we examine the behaviors of the dynamic gating unit, the main element of the model.

A gating vector, *z*∈[0, 1], is introduced. As *z* → 1, the model prefers to use features for gate generation. When *z* → 0, the model prefers structural features. We analyse gating values from 50 code samples in the test set and observe clear complexity adaptation.

In support of basic reasoning: For example, categorized in category “extremely short execution time,” the average gating value is relatively low (*z*≈0.32). This indicates that the simpler a piece of code, for example, simple assignments, getters/setters or math operations with no loops, the less of need for an extensive semantic interpretation of the code. The model directly uses information from the 16 handcrafted features. Therefore, the model is efficient and consistent with experts' intuitively correct observations on straightforward code.

### 5.2 Boundaries of static analysis

In support of intricate reasoning: for example, categorized in category 3, very long execution time, the average gated value increases significantly (*z*≈0.71). In situations where recursive or highly complex API invocations, for example, qsort, are used, or a large number of nested control statements exist, common performance analysis techniques based on basic statistical measures, such as loop depth, typically do not accurately reflect the performance problems, for instance, recursive functions report that they do not have a loop depth. Therefore, in these instances, the GFTrans model independently increases the weight carried by the vectors, thereby identifying some of the underlying performance problems. The accuracy of GFTrans, which is based entirely on static analysis techniques, at an accuracy level of 78.64% indicates that while GFTrans can produce accurate results with respect to the C programming language through static analysis, certain limitations exist. Based upon our assessment of the errors generated by GFTrans, two areas are identified as being problematic during our review of the error instances.

- **Pointer aliasing and dependency disruption:** pointer aliasing often causes GFTrans to fail at accurate data flow analysis. If a program has multi-level pointers, such as **p, pointer arithmetic, or complex memory transformations, static tools struggle to predict pointer targets before execution. This interrupts the “define-use” chains in the DTG. As a result, anchor tags are not set in the sequences. This results in the loss of crucial data flow context within the program. Such complexity can be underestimated. For example, a process running O(N) over a large array accessed via pointers may be labeled O(1).
- **Class confusion:** from the confusion matrix, we see that over 65% of wrong classifications are between adjacent categories, like mistaking category 1 for category 2. This happens because GFTrans uses fixed cutoffs for runtime labels. These cutoffs do not capture the true continuum of code complexity. Small code changes near a cutoff can change the model's classification result.

### 5.3 Practical

Conventional coding performance measures rely on dynamic profiling tools, such as Intel VTune (Intel, Santa Clara, CA, USA). These tools need developers to create test cases, compile the project, and sample data. This process often takes several minutes, depending on data coverage. In contrast, GFTrans provides a pre-emptive analysis. On the NVIDIA RTX 3090 (NVIDIA, Santa Clara, CA, USA), it takes an average of 47 ms to analyse a function. This includes graph extraction, feature calculation, and model inference. Quantised models work in the development environments. GFTrans's millisecond speed enables integration into IDEs as a syntax checker. GFTrans predicts performance class as developers code and flags in red when a function is likely “category 3, long-running.” This feedback lets developers tune performance before testing. It reduces refactoring debt later in the software lifecycle.

### 5.4 Performance across CPU architectures

We demonstrate that GFTrans is robust across different hardware. GFTrans learns the algorithmic time complexity [like *O*(*N*) vs. *O*(*N*^2^)]. A complex nested loop is algorithmically slow on any CPU. A simple assignment is fast on any CPU. While absolute time (nanoseconds) differs between Intel and AMD, the relative order stays the same. A sorting function is always slower than a math function. To verify the robustness of GFTrans on different hardware, we conducted a new experiment. We employed a machine with an AMD Ryzen 9 5900X CPU (AMD, Santa Clara, CA, USA). We took the original source code (2,887 functions). We re-ran all of them on the AMD machine. We collected new runtime labels specific to this CPU. We re-executed these codes on the AMD CPU to get new ground truth labels. Then, we used the GFTrans model to predict their categories. The results are shown in Table 7 below. 96.2% of the runtime labels remained the same on both CPUs. The broad category bins (e.g., 20–100 ms) absorb the minor speed differences. The accuracy on the AMD dataset is 77.92%. This is very close to the accuracy on Intel (78.64%). The performance drop is negligible (<0.8%). This proves that GFTrans generalizes well to AMD processors.

**Table 7:** Comparison of model performance on different hardware architectures.

**Hardware environment**	**Data source**	**Accuracy (%)**	**Macro-F1 (%)**
Intel Core i7-12700H (Baseline)	Collected on Intel	78.64	76.85
AMD Ryzen 9 5900X (Replication)	Collected on AMD	78.55	76.72

## 6 Conclusion

This work creates a new analysis framework, GFTrans, to predict performance without actually running the code, utilizing data anchor markers placed strategically within the control flow section of the code. Data anchor markers attach statistics from dynamic execution logic for each control flow path to the failure data dependencies for each control flow path, thereby allowing for statistical performance verification of the code. Also, by implementing a gating mechanism, GFTrans is capable of integrating both semantic attributes and manually created features into the evaluation data that enhance performance prediction. Experimental results indicate that GFTrans achieves an overall accuracy of 78.64% for real world datasets which exceeds the performance of the best baseline models and thus validates the usefulness of the graph structure fusion as a technique for achieving improved precision of performance predictions. GFTrans uses static analysis to provide real-time (milliseconds) performance feedback without compilation or execution. The mechanisms of GFTrans allow developers to tune their code for proper performance prior to merging code changes into their repositories, therefore, moving performance diagnostics from cumbersome testing phases to “coding-time” phases and significantly decreasing the amount of maintenance cost and refactoring cost incurred in the later phases of software development life cycles. In future work, we plan to extend GFTrans to support higher-level languages such as Python and Java. However, this expansion presents significant technical challenges. For dynamic languages like Python, execution time is heavily influenced by the interpreter's overhead, the Global Interpreter Lock (GIL), and dynamic typing, which are difficult to predict via static analysis alone. Similarly, for Java, the Just-In-Time (JIT) compilation mechanism performs dynamic optimizations during execution, meaning that the static control flow graph may not fully reflect the actual runtime behavior. Addressing these discrepancies will require incorporating runtime environment modeling into our static analysis framework.

## Data Availability

The dataset used in this study is not publicly available because the source code was collected from multiple open-source repositories whose authors do not permit commercial use or secondary redistribution of derived datasets. To respect the original authors' usage intentions and licensing constraints, the processed dataset cannot be released publicly. Access may be considered upon reasonable request to the corresponding author for academic and non-commercial purposes. Requests to access the datasets should be directed to YunBao Wen, 2023024289@m.scnu.edu.cn.
